# Capturing differences in perception and aesthetic judgment of live or medially presented music: development of a self-report instrument

**DOI:** 10.3389/fpsyg.2024.1339168

**Published:** 2024-04-02

**Authors:** Larina Sue Meinel, Claudia Bullerjahn, Alexander Lindau, Melanie Wald-Fuhrmann

**Affiliations:** ^1^Department of Social Sciences and Cultural Studies, Institute of Musicology and Music Education, Justus-Liebig-University Giessen, Giessen, Germany; ^2^Berliner Hochschule für Technik, Berlin, Germany; ^3^Department of Music, Max Planck Institute for Empirical Aesthetics, Frankfurt, Germany

**Keywords:** music perception, aesthetic judgment, live music, recorded music, audio-visual, 3D simulation, repertory grid technique, semantic differential

## Abstract

Nowadays there are multiple ways to perceive music, from attending concerts (live) to listening to recorded music through headphones (medial). In between there are many mixed modes, such as playback performances. In empirical music research, this plurality of performance forms has so far found little recognition. Until now no measuring instrument has existed that could adequately capture the differences in perception and aesthetic judgment. The purpose of our empirical investigation was to capture all dimensions relevant to such an assessment. Using 3D-simulations and dynamic binaural synthesis, various live and medial situations were simulated. A qualitative survey was conducted at the Department of Audio Communication of the Technical University of Berlin (TU Berlin). With the help of the repertory grid technique, a data pool of approximately 400 attribute pairs was created and individual rating data were collected. Our first study served to create a semantic differential. In a second study, this semantic differential was evaluated. The development of the semantic differential was carried out by first using a mixed-method approach to qualitative analysis according to grounded theory. Thereafter, a principal component analysis reduced the attribute pairs to 67 items in four components. The semantic differential consists of items concerning acoustic, visual and audio-visual interaction as well as items with an overarching assessment of the stimuli. The evaluation study, comprising 45 participants (23 male and 22 female, *M* = 42.56 years, *SD* = 17.16) who rated 12 stimuli each, reduced the items to 61 and resulted in 18 subscales and nine single items. Because the survey used simulations, the social component may be underrepresented. Nevertheless, the questionnaire we created enables the evaluation of music performances (especially for classical concerts) in a new scope, thus opening many opportunities for further research. For example, in a live concert context, we observed not only that seating position influences the judgment of sound quality but also that visual elements influence immersion and felt affect. In the future, the differential could be reviewed for a larger stimulus pool, extended or used modularly for different research questions.

## 1 Introduction

Listening to music is one of the ways humans like to spend their free time (Schäfer and Sedlmeier, 2018). Before Thomas Alva Edison invented the phonograph in 1877 – thus making it possible to reproduce music for the first time – attending a live performance was the only way to listen to music (Rocholl, 1976; Elste, 1992). Nowadays opportunities to perceive music are manifold. Listening to music has even become that easy, that in everyday life, music listening is mostly accompanied by another activity (Greb et al., 2018). However, studies which address the different performance forms in which people deliberately listen to music are rare (Wald-Fuhrmann et al., 2021). An accurate categorization of the presentation mode is additionally complicated by the growing plurality of performance forms. A recording may be listened to via headphones or mobile devices, an old radio set or a modern surround-system (aural). During music listening, people might also watch moving images such as in a music video or a television show (audio-visual) with the images showing details of the musicians’ performance (documentary) or visual material unrelated to the performance as such (structural, expression related, narrative or associative; see Bullerjahn and Hantschel, 2018). A live performance can take place without any technical support (unplugged); the musicians might use microphones (amplified); or the whole acoustic part of a performance might even be a reproduction of an audio file while the artists pretend to perform live with all typical facial expressions, gestures and postures (playback). Hence, the gradations are various and a breakdown of all relevant differences in music perception, experience and aesthetic judgment is so far missing (Lindau, 2010). We aim to close this gap with our studies and provide an instrument for future research on that topic. With an explorative approach, we want to discover and classify as many assessment dimensions as possible, but still we had to set priorities in order to not get to many variables. Hence, in the presented studies there is a great variety of different auditory simulations but fewer variations of visual situations. Depending on the kind of music used in future studies there may be some items omitted (e.g., stimuli without visual component) or have to be extended by other factors (e.g., because of narration due to text or music video). We want to provide a basis for all the future research that hopefully will follow in this area.

As early as 2001, Finnäs stated in a review of empirical research that most studies insufficiently report and evaluate the presentation modes, or merely refer to them in terms of contrasting pairs, such as “live vs. medial” or “aural vs. audio-visual.” Although in recent years it has become more common for musical research to report the presentation mode more properly, the other criticized aspects have not substantially changed in the past 20 years.

We occasionally find comparisons between live and medial music in the field of consumer research. Rondán-Cataluña and Martín-Ruiz (2010) examined the development of the income generated by CDs and sales of concert tickets and contrasted it with customers’ perceptions about concerts and CDs. Mortimer et al. (2012) dealt with the endangered profitability of recorded music due to filesharing and examined whether this affects live concerts. Both studies were unable to find any negative effect on income from concerts, but did find decreasing prices for recorded music. Paradoxically, “consumer satisfaction, price fairness perception, willingness to pay, customer value, and product/service quality are significantly more highly ranked in concert attendees than in CD buyers” (Rondán-Cataluña and Martín-Ruiz, 2010, p. 1410).

Roose and Vander Stichele (2010) examined the composition of groups of people who prefer listening to live music (“public”) or recordings (“private”). It turned out that music consumption was in principle positively related to all indicators of cultural capital (with strongest effects for high-brow genres), but cultural capital was more important for attending live concerts of any given music genre than for listening to recordings at home. Somewhat unexpectedly, they did not find any effect of income. Coutinho and Scherer (2017) compared emotions induced by the same performance of three German Lieder (art songs) in varied listening contexts. Listeners of a Lieder recital in a church showed extremely consistent emotional responses and reported significantly more feelings of wonder, while listeners of an audio-visual recording in a university lecture hall reported significantly more boredom. Brown and Krause (2020) found that while digital files provide their users with control over their own music and the listening place and time, live music is preferred because of its authenticity, social aspects and its ability to stir strong feelings.

Brown and Knox (2017) identified that the experience of presence in a unique event with like-minded people and the uncertainty as to whether the performances will be either as anticipated, novel or disappointing were primary motivations behind live music attendance (see also Pitts, 2014). Swarbrick et al. (2019) used motion capture of head movements given that these often reflect emotions as well as the affiliated social engagement experienced among people moving together to music. They compared the movement responses of audiences attending a live concert with the rock artist present to audiences listening to a concert with the artist absent but with the same songs played from a recorded, but not yet released album. During the live concert, head movements were faster and more entrained than during the album-playback concert and they were fastest and most entrained for self-reported fans of the artist. The results indicate that live music leads to a greater engagement for listeners than recorded music, and this is especially so in the case of admiration for the artist, demonstrating the creation of a unique bond between fan and performer while controlling for the effects of togetherness in an audience.

There also exists some research about the medical or educational use of live music in contrast to aurally presented medial music. Thus, Flink (1990) determined a slightly higher will of patients to participate actively and spontaneously in a music therapy session if music was presented live; and Shoda et al. (2016) discovered a greater entrainment of the heartbeats of an audience with the musical rhythm and greater relaxation at a live performance than when listening to recordings.

Research comparing medial music with and without a visual component is more common. Even though there are most likely even more studies about this topic by now (e.g., Vuoskoski et al., 2014, 2016; Coutinho and Scherer, 2017; Li et al., 2021), there are good overview articles by Platz and Kopiez (2012), which features a meta-analysis of 15 aggregated studies on audio-visual music perception, and Finnäs (2001), which presents a systematic review of now dated empirical research in which listening to the same music was compared under different conditions of presentation (live, audio-visual, aural). The main findings of these examinations are that visual appearance has an important influence in the communication of meaning and therefore on the judgment of music. Platz and Kopiez (2012) detected an average medium effect size of 0.51 standard deviations (Cohen’s *d*; 95% CI [0.42, 0.59]) for the visual component. The judgment of the music depends on the visual material. Sometimes the music was rated as more likable without the visual component, sometimes it was the other way round. In particular, performance quality is substantially perceived and judged by the way of performer movements, even in the absence of any auditory information pointing to a substantial effect of performers’ movements and gestures (Griffiths and Reay, 2018). Furthermore, averaged electrodermal activity– representing felt arousal of listeners – was significantly higher in an audio-visual presentation of Igor Stravinsky’s second piece for solo clarinet, as compared with audio-only and visual-only presentations (Chapados and Levitin, 2008). This suggests that an audio-visual presentation possesses an “emergent property” of its own created by the bimodal interaction.

One of the few studies comparing different types of aural presentation modes (Lipscomb and Kerins, 2004) examined the influence of two-channel stereo vs. 5.1 surround sound in the cinematic and music listening experience. The results reveal that presentation mode was a negligible factor in music listening compared with viewing a movie with synchronized music. It appeared that especially, participants with higher levels of visual training were influenced by the presentation mode in their verbal responses.

What these previous studies all share is that they emphasize very few presentation modes and focus only on selected perceptual differences. The aim of this study, then, was to identify all relevant perceptual differences and solidify them in the form of a semantic differential. This includes acoustic as well as visual perception. Despite this, the semantic differential should also capture dimensions that can either influence or result from perception and experience such as aesthetic judgment. In future, then, the relationship of these different factors can be examined using the same questionnaire. In preparation for this study, Lindau (2010) published the results of an online-survey study in which participants could freely attempt to predict the difference dimension of live or medially presented music. Tonal aspects were mentioned most often (including localization, dynamic range, loudness, acoustic and timbre). The other categories were emotionality, perfection, sociality, the senses (including visuality), repeatability, ubiquity, immediacy, control and attention. To survey the differences in the perception, experience and aesthetic judgment in this study, a method was chosen by which the participants could compare different simulations of live or medial presentation forms and thereby develop a vocabulary for describing their differences. To create this semantic differential, two studies were conducted. The first study served the creation of the semantic differential and the second served the evaluation and finalization. It is the continuation of a project that was started at the Technical University of Berlin (TU Berlin; Horn et al., 2015). The production of the stimuli and the data collection took place solely at the TU Berlin. When it came to the analysis, the research institutions in Giessen and Frankfurt/Main took over.

## 2 Study 1: Creation of the questionnaire

### 2.1 Materials and methods

#### 2.1.1 Participants

Starting point of the data collection was the personal construct theory of George Alexander Kelly (1991), which says that people use personal constructs of opposing poles to explain how they see the world. The repertory grid technique is a qualitative method for detecting these personal constructs and can thus be used for participant numbers as low as one (Kelly, 1991; Rosenberger and Freitag, 2009). For this study, not only the view of one person, but also a large database with attributes describing all kinds of differences in the perception, experience and aesthetic evaluation of live or medially presented music was to be generated. Therefore, participants were chosen who dealt intensively with music either professionally or privately. This allowed us to assume that the sample consisted of differentiating listeners. Due to the diversity of their accesses to music (for example, passionate visitor of concerts, sound engineer or music theorist) the listeners generated a diverse vocabulary.

The sample comprised multiple strata with regard to age (young adults, middle-aged and elderly people), gender (female and male) and musical background (professional musicians, professional sound engineering background and private music or media enthusiastic) so that a total number of 3 × 2 × 3 = 18 subjects between 20 and 66 years of age participated (9f, 9 m, *M* = 42.56 years, SD = 17.16). For the purpose of this study and the envisaged methods, this was an appropriate number of participants. With a sufficient number of stimuli, some similar or identical attributes are already assigned from participant two onwards, so that with 18 people it is highly likely that all important differences have been named and it is also possible to recognize what the most common differences are. The thus stratified participants were assigned randomly to three conditions: aural, audio-visual I and audio-visual II. Table 1 shows the distribution of participants per condition.

**Table 1:** Participants in each condition: number (*n*), mean (*M*) with standard deviation (SD) and gender.

	*n*	Age		Gender	
Condition		*M*	SD	Male	Female
Aural	7	43.14	16.69	4	3
Audio-visual I	6	45.33	16.74	3	3
Audio-visual II	5	38.40	21.22	2	3

#### 2.1.2 Simulation environment and stimuli

For the elicitation of the differences in perception, experience and judgment, a simulation environment was created which could – due to binaural synthesis – dynamically simulate both live and medial perception situations. For this study it would not have been expedient to use an actual live concert as it was necessary to have full control over the acoustic parameters (e.g., without interfering noises of other listeners or the musicians themselves). Furthermore, the participants had to be able to watch the stimuli repeatedly and switch between different listening situations. With this kind of acoustic simulation, it has been shown that listeners cannot differentiate between real or simulated sources of sound (Moldrzyk et al., 2005; Maempel, 2008).

The visual simulation was created by stereoscope video recordings presented on a big screen as 3D videos. Different perception situations were imitated, from listening to music through a loudspeaker to a live concert performance. Different acoustic and different visual perception situations were simulated, but the focus was on acoustic factors. Detailed descriptions of the simulation environment and the production of the stimuli can be found in Horn (2013), Horn et al. (2015), and Lindau (2014).

Stimuli were produced for two different pieces of music. One stimulus was the first movement of Wolfgang Amadeus Mozart’s String Quartet No. 1 in G major (KV 80), which was recorded by the Reinhold Quartett and presented by the Berliner 3plus1 Quartett in playback. The other stimulus was the Tango “Chantey “by Thomas Zaufke, with the same string quartets as well as voice recorded and presented by Yamil Borges. These music pieces were chosen for different reasons. As there were no existing available stimuli which were free of reverb and of high quality, the stimuli had to be newly produced for the study. The number of instruments involved was to be technically, organizationally and financially acceptable. Furthermore, sheet music had to be available for the instrumentation, and the same group of performers (plus voice for the second piece) need to be able to perform with as much stylistic variation as possible. Lastly, one of the music pieces was to be only instrumental and the other was to have a vocal component in order to vary the stimuli as widely as possible.

**Figure 1:**
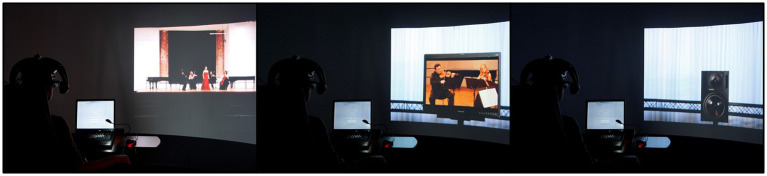
The three different audio-visual presentation modes “live,” “TV screen with documentary video” and “loudspeaker” in the simulation environment.

In the condition “aural,” the participants did not see anything at all but just listened to the acoustic simulations of the different presentation modes. The groups with the conditions “audio-visual I” and “audio-visual II” all listened to the same acoustic simulations and watched the corresponding visual 3D-simulations of live performances and medial presentations, but the latter were either the 3D-simulations of a TV screen with documentary videos of the musicians performing in a concert (audio-visual I) or the 3D-simulations of one or more loudspeakers (audio-visual II) (*cf.* Figure 1). The documentary videos showed the performers in a sequence of different shot sizes.

**Figure 2:**
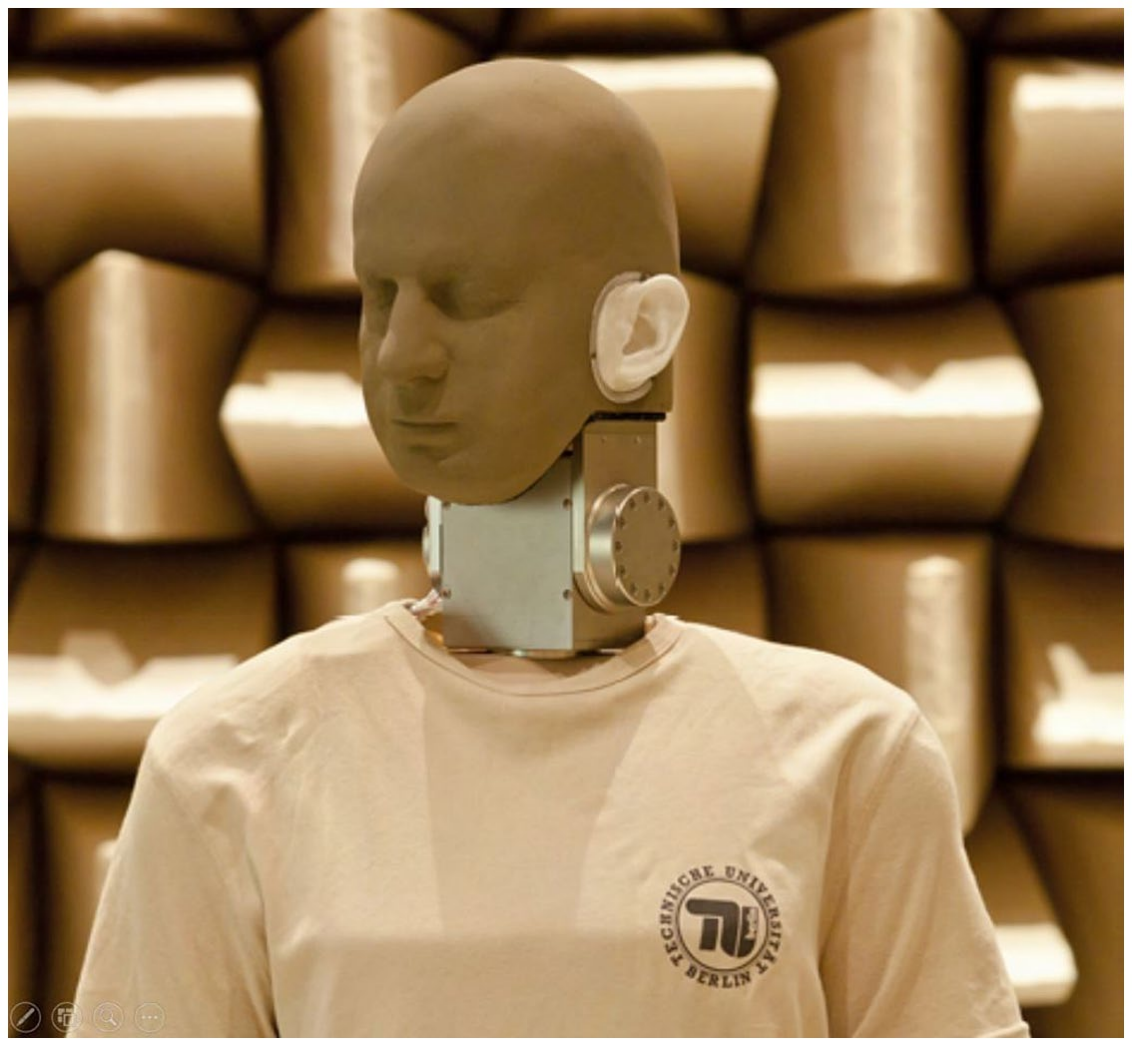
The binaural measuring robot (Horn, 2013, p. 26).

In total, the participants watched and listened to 28 different stimuli, divided into 14 different acoustic simulations per piece of music: one simulation from a seat in front at a live concert, one simulation from a seat in the rear, once each with and without motion-tracked binaural (MTB) background noise of a concert hall; one mono-simulation, three stereo-simulations from the studio and two more stereo-earphone-simulations; two surround recordings from the studio and at least two recordings with wave field synthesis (WFS). So there were four live simulations and ten media simulations per piece of music. With the large number of stimuli, it should be ensured that even small differences between different recording techniques can be detected. The frequency characteristics correspond to merchantable setups of a sound engineer. Recordings which were free of reverb were produced using microphones of the company Neumann (e.g., Neumann KM140) and subsequently used to produce the live and media stimuli with the help of a binaural measuring robot (*cf.* Figure 2). The sound pressure level for the media stimuli was set up by an expert-based manufacturing process, as the volume of media stimuli could be chosen individually by the listeners. All stimuli had a length of approximately 1 min and 16 s. A more detailed list of the audio-visual presentation modes can be found in Table 2.

**Table 2:** Audio-visual presentation modes with different combinations of simulated listening situation and implemented audio-recording technology.

No.	listening situation	Audio recording technology	Condition
1	Front seating at a live concert	With MTB	Audio-visual I & audio-visual II
2	Front seating at a live concert	Without MTB	Audio-visual I & audio-visual II
3	Rear seating at a live concert	With MTB	Audio-visual I & audio-visual II
4	Rear seating at a live concert	Without MTB	Audio-visual I & audio-visual II
5	Documentary video on TV screen	Omnidirectional (mono loudspeaker)	Audio-visual I
6	Documentary video on TV screen	AB (spaced microphone stereo)	Audio-visual I
7	Documentary video on TV screen	XY (coincidental stereo)	Audio-visual I
8	Documentary video on TV screen	ORTF (stereo loudspeaker)	Audio-visual I
9	Documentary video on TV screen	Decca (surround loudspeaker)	Audio-visual I
10	Documentary video on TV screen	INA-3 (surround loudspeaker)	Audio-visual I
11	Documentary video on TV screen	AB (headphones)	Audio-visual I
12	Documentary video on TV screen	XY (headphones)	Audio-visual I
13	Documentary video on TV screen with additional loudspeakers in the background	WFS (conventional)	Audio-visual I
14	Documentary video on TV screen with additional loudspeakers in the background	WFS (creative)	Audio-visual I
15	Loudspeaker	Omnidirectional (mono loudspeaker)	Audio-visual II
16	Loudspeaker	AB (spaced microphone stereo)	Audio-visual II
17	Loudspeaker	XY (coincidental stereo)	Audio-visual II
18	Loudspeaker	ORTF (stereo loudspeaker)	Audio-visual II
19	Loudspeaker	Decca (surround loudspeaker)	Audio-visual II
20	Loudspeaker	INA-3 (surround loudspeaker)	Audio-visual II
21	Loudspeaker	AB (headphones)	Audio-visual II
22	Loudspeaker	XY (headphones)	Audio-visual II
23	Several loudspeakers	WFS (conventional)	Audio-visual II
24	Several loudspeakers	WFS (creative)	Audio-visual II

All combinations were applied to both pieces of music and were presented in mixed order. MTB = motion-tracked binaural background noise of a concert hall; WFS = wave field synthesis; audio-visual I = live simulation and documentary video; audio-visual II = live simulation and loudspeaker.

#### 2.1.3 Procedure

The study took place in a room that was constructed for sound production, which is why an extremely low level of background noise can be assumed. All participants were tested in single sessions. The participants were placed in the simulation environment and they additionally had a computer on which they could autonomously rate the stimuli. The stimuli were presented in groups of three (so called triads) and rated by the participants using the repertory grid technique. Participants could switch between the three stimuli at will. In the condition “aural,” participants heard audio only, while in the conditions audio-visual I & II, participants both heard the audio and watched the associated 3D-simulation. They had to mention the contrast of one stimulus compared to the other two and subsequently supplement the contrast with an opposite pole so that the attribute pair was completed. The participants could fill in their answers in the text field provided for this purpose on the computer.

A total number of 18 triads was presented to each participant. Each triad could be repeated *ad libitum* and any number of contrasting attributes per triad could be created.

Finally, the participants rated all 28 stimuli with their own constructs (= the contrasting attribute pairs) on seven-level scales. In this way, 18 single grids were created with 12–30 constructs per person. On the whole, there was a total number of 376 attribute pairs (cf. Supplementary material). In addition, the investigator documented when the participants gave comments to their constructs.

### 2.2 Results

For the analysis, a mixed-methods design was chosen due to the mixture of qualitative and quantitative data. While the rating can be used for structural models, the inter-individual summary of the attributes from the different people can only take place on a qualitative basis.

The analysis was divided into three steps. In the first step all constructs were categorized with the grounded theory methodology to receive a first overview concerning the dimension of the different presentation forms and, meanwhile, create a basis for the following steps. According to the grounded theory, the constructs were coded in several runs beginning very close to the text. Afterwards the constructs were grouped little by little until only a few categories remained (Strauss, 1994). Four categories were identified: acoustic, visual, the interaction of acoustic and visual and general assessment. The acoustic category formed the largest group.

In the second step the single grids were used for explorative factor analyses – more detailed principal component analysis (PCA) with oblique rotations. This helped to structure the data and find redundancies. The PCAs were performed based on the categories from step one. In this way, attributes which were clearly separated in terms of content (like acoustic and visual) were not analyzed together, which would have led to misleading results. The number of factors was determined by the Kaiser-Guttman criterion. This method tends to identify more factors than the other criteria, which was suitable for that step of the analysis. For all factors there was also calculated the reliability value Cronbach’s Alpha for different factor compositions. In this step, no attributes were removed, as the aim was to discover intersubjective relationships and not the factor structures of individuals.

The results of the PCAs served as a basis of interpretation for the qualitative summary in an intersubjective questionnaire. The single grids were also consulted for this step. In the summary, redundant attribute pairs of different participants were to be eliminated, with the most common formulations remaining intact. The summary took place following the qualitative content analysis of Philipp Mayring (2015). Theoretical foundations were included, mostly in order to secure intersubjective understanding. For example, trivial (like *to hear and see: musicians perform in a room* – *to hear and see: recording comes from loudspeaker*); undifferentiated (like *concert situation [spatial] – typical CD-recording*, which mixed an attribute that is formulated fuzzy with a purely acoustic description); or attributes that were potentially hard to understand, eliminated or revised. It was often impossible to clearly assign such items in the PCAs in advance. Formulations used by many participants were retained, as were items which were not named often but that addressed aspects that did not occur with other participants. The remaining items were to include all addressed aspects, form clear opposites, be understandable and be applicable for a wide range of different stimuli.

The thus-formed semantic differential consists of 33 acoustic attributes, 12 visual attributes, six attributes of the interaction of acoustic and visual and 17 attributes of general assessment (*cf.* Table 3).

**Table 3:** The preliminary semantic differential.

German original	English translation
Bewerten Sie, was Sie gehört haben!	Rate what you have heard!
Musizierende unterschiedlich weit entfernt – Musizierende in gleicher Entfernung	Musicians at different distances – musicians at same distance
Stimmen nicht ausgeglichen – gute Balance der Stimmen	Voices not balanced – good balance of the voices
Einzelne (Instrumental-)Stimme(n) im Vordergrund – Einzelne (Instrumental-)Stimme(n) im Hintergrund	Single (instrumental) voice(s) in the foreground – single (instrumental) voice(s) in the background
Hauptstimme überrepräsentiert – Hauptstimme unterrepräsentiert	Main voice overrepresented – main voice underrepresented
Mono – Stereo/Surround	Mono – stereo/surround
Trocken – hallig	Dry – reverberant
Schmales Panorama – breites Panorama	Slim panorama – wide panorama
Kleiner Raum – großer Raum	Small room – large room
Instrumente schlecht zu orten – Instrumente gut zu orten	Instruments can be poorly localized – instruments can be well localized
Transparent – undurchsichtig	Transparent – obscure
Geringe Tiefenstaffelung – große Tiefenstaffelung	Low depth staggering – high depth staggering
Laut – leise	Loud – quiet
Nah – entfernt	Near – distant
Klang von links – Klang von rechts	Sound from the left – sound from the right
Musik spielt im Kopf – Musik spielt im Raum	Music plays in the head – music plays in the room
Klang von oben – Klang von unten	Sound from the top – sound from the bottom
Dünner Klang – voller Klang	Thin sound – full sound
Frequenzen nicht ausgeglichen – Frequenzen ausgeglichen	Frequencies not balanced – frequencies balanced
Kalt – warm	Cold – warm
Hart – weich	Hard – soft
Hell – dunkel	Bright – dark
Tiefen dominieren – Höhen dominieren	Lows dominate – highs dominate
Schrill – sonor	Shrill – sonorous
Kontrolliert – unbändig	Controlled – unrestrained
Leblos – lebhaft	Lifeless – lively
Uninspiriert – geistvoll	Uninspired – spirited
Leicht – angestrengt	Easy – strained
CD-Qualität – Amateuraufnahme	CD-quality – amateur recording
Kofferradioklang – guter Klang	Sounds like a portable radio – good sound
Blechern – nicht blechern	Tinny – full
Bearbeitet – natürlich	Edited – natural
Authentisch – künstlich	Authentic – artifical
Steril – voll Leben	Sterile – full of life
Bewerten Sie, was Sie gesehen haben!	Rate what you have seen!
Kaltes Licht – warmes Licht	Cold light – warm light
Bläuliche Farben – gelbliche Farben	Bluey colors – yellowish colors
Farblich abwechslungsreich – farblich gleichförmig	Varying colors – steady colors
Zurückhaltende Gestik – übertriebene Gestik	Reserved gestures – exaggerated gestures
Statisches Bild – bewegtes Bild	Static picture – moving picture
Bild zurückgenommen – Bild unterhaltsam	Reticent picture – entertaining picture
Künstliche Umgebung – natürliche Umgebung	Artifical environment – natural environment
Involvierte Ausstrahlung der Musiker – Musiker wirken distanziert	Involved aura of the musicians – distanced aura of the musicians
Musiker wirken verloren – angemessene Darstellung der Musiker	Musicians appear lost – musicians are presented appropriately
Interaktionen gut sichtbar – Interaktionen schlecht sichtbar	Interactions clearly visible – interactions poorly visible
Kleiner Bildausschnitt – großer Bildausschnitt	Small shot size – big shot size
Entfernt – nah	Distant – near
Bewerten Sie das Zusammenspiel von Bild und Ton!	Rate the interaction between image and sound!
Mischung (Klang) ist bildbezogen – Mischung (Klang) lässt Bild außen vor	Mix (sound) is adjusted to the image – mix (sound) ignores the image
Durch Bild erzeugte Erwartungen werden erfüllt – durch Bild erzeugte Erwartungen werden nicht erfüllt	Expectations which are created by the images are fulfilled – expectations which are created by the images are not fulfilled
Man glaubt mehr Musiker zu hören als da sind – man hört die Originalbesetzung exakt	One seems to hear more musicians than there actually are – one can hear the exact number of musicians
Zusammenspiel von Bild und Ton verwirrt – Zusammenspiel von Bild und Ton ist hilfreich	The interaction between image and sound confuses – the interaction between image and sound clarifies
Ton passt gut zum Bild – Ton passt schlecht zum Bild	Sound fits well with the image – sound fits poorly with the image
Optisch und akustisch unterschiedlich weit entfernt – optisch und akustisch in gleicher Entfernung	Visually and acoustically at different distances – visually and acoustically at the same distance
Bewerten Sie das gezeigte Material allgemein!	Rate the shown material in general!
Überzeugt mich nicht – reißt mich mit	Unsatisfactory – enthralls me
Wenig einbezogen – zieht mich rein	Does not pull me in – pulls me in
Fühle mich vor Ort – fühle mich außen vor	I feel like I am there – I feel far away
Distanziert – involviert	Reserved – involved
Privat – öffentlich	Private - public
Ambiente wie großes Konzert – Ambiente wie Probedurchlauf	Ambiance of a huge concert – ambiance of a test rehearsal
Bewirkt intensives Zuhören – wirkt wie Hintergrundmusik	Causes intense listening – seems like background music
Schwächlich – mächtig	Weak – powerful
Gemütlich – stressig	Cozy – stressful
Beruhigend – aufwühlend	Calming – stirring
Reizvoll – langweilig	Appealing – boring
Angenehm – unangenehm	Pleasant – unpleasant
Geht direkt in die Seele – gefühlsneutral	Touches the soul – neutral feelings
Wirkt auf mich emotional intensiv – erreicht mich emotional kaum	Evokes intense emotions – does not reach me emotionally
Gefällt mir – gefällt mir nicht	I like – I dislike
Würde ich kaufen – würde ich nicht kaufen	I would buy it – I would not buy it
Schön – hässlich	Beautiful – ugly

### 2.3 Discussion

The semantic differential now consists of 68 items, leaned on the common parlance of the participants. This is a quiet long differential that would benefit of an examination and improvement of the structure. As no-one used the complete differential so far it is useful to evaluate it with a new sample. The participants of the first stratified sample were at least music enthusiast, so the new participants should be of varying musical expertise.

Further application areas and limitations are discussed in an overarching discussion at the end of this research report.

## 3 Study 2: Evaluation of the questionnaire

The second study served for the evaluation and finalization of the semantic differential. The aim was to eliminate items which are not understandable for the participants or redundant, to identify subscales and to revise the questionnaire according to the findings. In addition, the questionnaire was checked for any correlations between the understanding of the questionnaire and the musical experience of the participants.

### 3.1 Materials and methods

#### 3.1.1 Participants

As the evaluation of the semantic differential was mainly quantitative, for this study a larger number of participants was needed. Due to the large amount of time required for the procedure in individual sessions with 17 repeatedly watched stimuli each (including four for familiarization and one twice), only a sample size of at least 30 was targeted, although a much larger sample size would certainly have been desirable. The participants were recruited from the participant database of the Max Planck Institute for Empirical Aesthetics. Inclusion criteria were: German mother tongue, majority age, ability to see three-dimensionally and intact hearing abilities (self-reported). The participants also ought not to have participated in the first study. The participants were compensated with 5€ per half an hour.

In total, 57 people filled out the questionnaire. After the data was checked for reliability based on a stimulus, which occurred two times, a total number of 45 participants remained. Due to diverse reasons (e.g., malfunction of the internet connection, conflict of appointments) some test subjects could not rate all stimuli, so one person rated eight, one person rated nine, one person rated 11 and two rated 12 stimuli (out of 13).

The remaining participants had a mean age of *M* = 38 years (*SD* = 16.14) ranging from 19 to 72 years. The number of men and women was nearly equal: 23 men participated (*M* = 42.57 years, *SD* = 15.79) and 22 women (*M* = 33.23 years, *SD* = 15.42). Out of 45 subjects, two had an intermediate school leaving certificate (“Mittlere Reife”), 21 a general matriculation standard or subject-related entrance qualification, six a bachelor degree, and 16 a university degree (e.g., diploma, master’s or state examination). Thus, the education was on a high level. To check for musical experience, the participants were asked to fill in the Goldsmith Sophistication Index (GOLD-MSI). This allowed us to determine whether the experience of the listeners had any influence on the estimation of the stimuli and the understanding of the attributes. The GOLD-MSI consists of 38 items on which the participants rate their musical experience via self-report in response to different statements concerning their interest in or connection with music. The GOLD-MSI is divided in the subscales “Active Engagement,” “Perceptual Abilities,” “Musical Training,” “Singing Abilities” and “Sophisticated Emotional Engagement with Music.” For this study, only the first three subscales were used (25 items). The average score of the GOLD-MSI was *M* = 113.22 (*SD* = 18.65) and therefore a little higher than the score Schaal et al. (2014) identified for a German sample (*n* = 641, *M* = 101.68).

#### 3.1.2 Stimuli

Twelve of the stimuli, which had already been used in the first study, served as stimuli for Study 2 (shortened to a length of 34–37 s). They were meant to cover a spectrum of different presentation modes that was as broad as possible. In contrast to the first study, the participants were not divided into different groups. All participants received the same 3D simulations which showed either loudspeakers or a TV screen presenting a documentary video of a concert or the 3D simulation of a live performance (*cf.* Figure 1). The acoustic simulations were varied, as in Study 1. To check for reliability, one stimulus was used twice. Table 4 shows a brief description of the chosen stimuli.

**Table 4:** Overview of the selected stimuli used in Study 2.

Stimulus number	Piece of music	Listening situation	Audio recording technology
1	Mozart	Front seating at a live concert	With MTB
2	Mozart	Documentary video on TV screen	Omnidirectional (mono)
3	Mozart	Documentary video on TV screen	AB (spaced microphone stereo)
4	Mozart	Loudspeaker	INA-3 (surround)
5	Mozart	Loudspeaker	XY (headphones)
6	Mozart	Documentary video on TV screen	WFS
7	Zaufke	Front seating at a live concert	With MTB
8	Zaufke	Rear seating at a live concert	With MTB
9	Zaufke	Loudspeaker	AB (spaced microphone stereo)
10	Zaufke	Documentary video on TV screen	Decca (surround)
11	Zaufke	Documentary video on TV screen	AB (headphones)
12	Zaufke	Loudspeaker	WFS
13 (reliability)	Mozart	Documentary video on TV screen	AB (spaced microphone stereo)

MTB = motion-tracked binaural background noise of a concert hall; WFS = wave field synthesis.

#### 3.1.3 Procedure

Before the survey began, the procedure was tested in a few preliminary tests.

The survey took place at the recording studio of the Max Planck Institute for Empirical Aesthetics in Frankfurt/Main, which is a room-in-room construction, so again there was hardly any background noise. The stimuli were presented on a 75-inch 3D TV and the sound was played through Beyerdynamic DT 990 pro headphones.^1^ As no motion-tracking system was in place, it was not possible to move the audio sources synchronously with the head movements; but hardly any head movements could be observed. Each participant was centered alone in a darkened room, but could be seen via a webcam by the investigator and was able to communicate through an intercom system. The duration of the survey was about 2 h. The participants had the option to take intermittent breaks, but only very few people made use of it.

The participants were instructed to sit down and make themselves comfortable in order to ensure a relaxed concert and media simulation. Furthermore, they were provided brief instructions in the handling of not only the tablet, which was used to fill in the questionnaire, but the 3D-TV and the intercom system as well. The mouse for controlling the TV was placed on either the left or the right side depending on the wishes of the participant.

Before the start of the actual study, four demonstration videos were presented to give an idea of the scope of the differences between the stimuli and to practice using the equipment. Then the stimuli were presented in a random order. Each stimulus was to be watched repeatedly and rated on a seven-level scale. As an additional option, the participants could also respond with “I cannot answer.” The items were randomized but remained sorted in the categories.

In addition to rating the stimuli, the participants also filled in the Goldsmiths Musical Sophistication Index (Schaal et al., 2014). Subsequently, some socio-demographic data was to be filled in as well.

#### 3.1.4 Design and analysis

The dataset for the evaluation consisted of 45 participants which delivered 13 judgments each (12 different stimuli + one duplicate for reliability check; within-subjects design with 13 repetitions of measurement). To analyze item difficulty, discriminatory power and scale reliability for each item, a value over all repetitions was aggregated. Furthermore, some variables for live and mediated stimuli were aggregated (cf. Supplementary material).

The significance level was set at α = 0.05.

### 3.2 Results

#### 3.2.1 Item analyses

*Item variance, missing values and item difficulty*: Initially, the means, standard deviation, variance and number of missing values (“I cannot answer”) was identified for all items via descriptive statistics. For all items, the whole scale was exploited. Items with 10 % or more missing values were checked concerning reasons induced by the stimuli (e.g., difficulties in identifying a “main voice” in the string quartet by Mozart) or clues to problems of comprehension (such as for “low depth staggering – high depth staggering” with 98 missing values out of 573, but no obvious pattern concerning certain stimuli). The difficulty of all items was between 0.37 and 0.72, which is on an acceptable level.

#### 3.2.2 Group comparisons

For each item an analysis of variance (ANOVA) for repeated measurements was performed in order to establish which items were useful for differentiating between different presentation modes. As it is not relevant for the aims of this study to detect the differences, no post-hoc tests were conducted at this point. Four items did not reach the significance level.

*Slim panorama – wide panorama* missed a significant result with *F* (12, 312) = 1.468 and *p* = 0.173. The check for missing values indicated that there might be problems in understanding, as there were 67 missing values.

The construct *sound from the top – sound from the bottom* did not reach the significance level [*F* (6.680, 167.003) = 1.610, *p* = 0.139]. Problems in understanding were nevertheless unlikely as the similar item *sound from the left – sound from the right* did not cause any problems.

The item *lows dominate – highs dominate* just barely missed the significance level with *F* (7.237, 246.047) = 1.871 und *p* = 0.072. For the musically more experienced participants, the significance level was reached with *F* (12, 192) = 2.070 und *p* = 0.021.

The last item that missed a significant result in the group comparisons was *one seems to hear more musicians than there actually are – one can hear the exact number of musicians*. With *F* (5.673, 204.235) = 1.050 und *p* = 0.393, it was clearly above the significance level, but it still could have been caused by the stimuli. Effects such as the overdub technique are much more common in the production of popular music (Maempel et al., 2008).

#### 3.2.3 Musical experience and Age

Overall, there were only three items that correlated with the musical experience: the attribute pairs *instruments can be poorly localized – instruments can be well localized* with *r* = 0.344 and *p* = 0.021, *one seems to hear more musicians than there actually are – one can hear the exact number of musicians* with *r* = 0.373 and *p* = 0.012 and *private – public* with *r* = −0.357 and *p* = 0.016. It is not surprising that people with increasing musical sophistication did rate the instruments as easily localizable and also claimed to hear the exact number of musicians due to their greater experience in listening to different music pieces. The greater feeling of private music listening could be attributed to the absence of an audience in the live-stimuli, which might have attracted more attention from people who are used to attending live performances. Age and musical experience do not correlate. There were several items that correlated with age. Interestingly there does not seem to be any influence of the hearing ability but the elderly people seem to have rated a little more negative than the younger participants. All items that reached significant correlations are shown in Table 5.

**Table 5:** Significant Item correlations with age, Item-name, Spearman‘s Rho (ρ) and *p*-value.

Item	*ρ*	*p*
Voices not balanced – good balance of the voices (Stimmen nicht ausgeglichen – gute Balance der Stimmen)	−0.322	0.031
Near – distant (Nah – entfernt)	−0.337	0.024
Artifical – authentic (Künstlich – authentisch)	0.377	0.011
Sound from the top – sound from the bottom (Klang von oben – Klang von unten)	0.352	0.018
Bright – dark (Hell – dunkel)	0.295	0.049
Reserved gestures – exaggerated gestures (Zurückhaltende Gestik – übertriebene Gestik)	−0.347	0.019
Static picture – moving picture (Statisches Bild – bewegtes Bild)	−0.420	0.033
Reticent picture – entertaining picture (Bild zurückgenommen – Bild unterhaltsam)	−0.384	0.009
Involved aura of the musicians – distanced aura of the musicians (Involvierte Ausstrahlung der MusikerInnen – MusikerInnen wirken distanziert)	0.473	0.001
Musicians appear present – musicans appear lost (MusikerInnen wirken präsent – MusikerInnen wirken verloren)	0.349	0.019
Unsatisfactory – enthralls me (Überzeugt mich nicht – reißt mich mit)	−0.328	0.028
Cozy – stressful (Gemütlich – stressig)	0.499	< 0.001
Calming – stirring (Beruhigend – aufwühlend)	0.521	< 0.001
Pleasant – unpleasant (Angenehm – unangenehm)	0.384	0.009
I like – I dislike (Gefällt mir – gefällt mir nicht)	0.398	0.007
Beautiful – ugly (Schön – hässlich)	0.453	0.002

#### 3.2.4 Scale development

**Figure 3:**
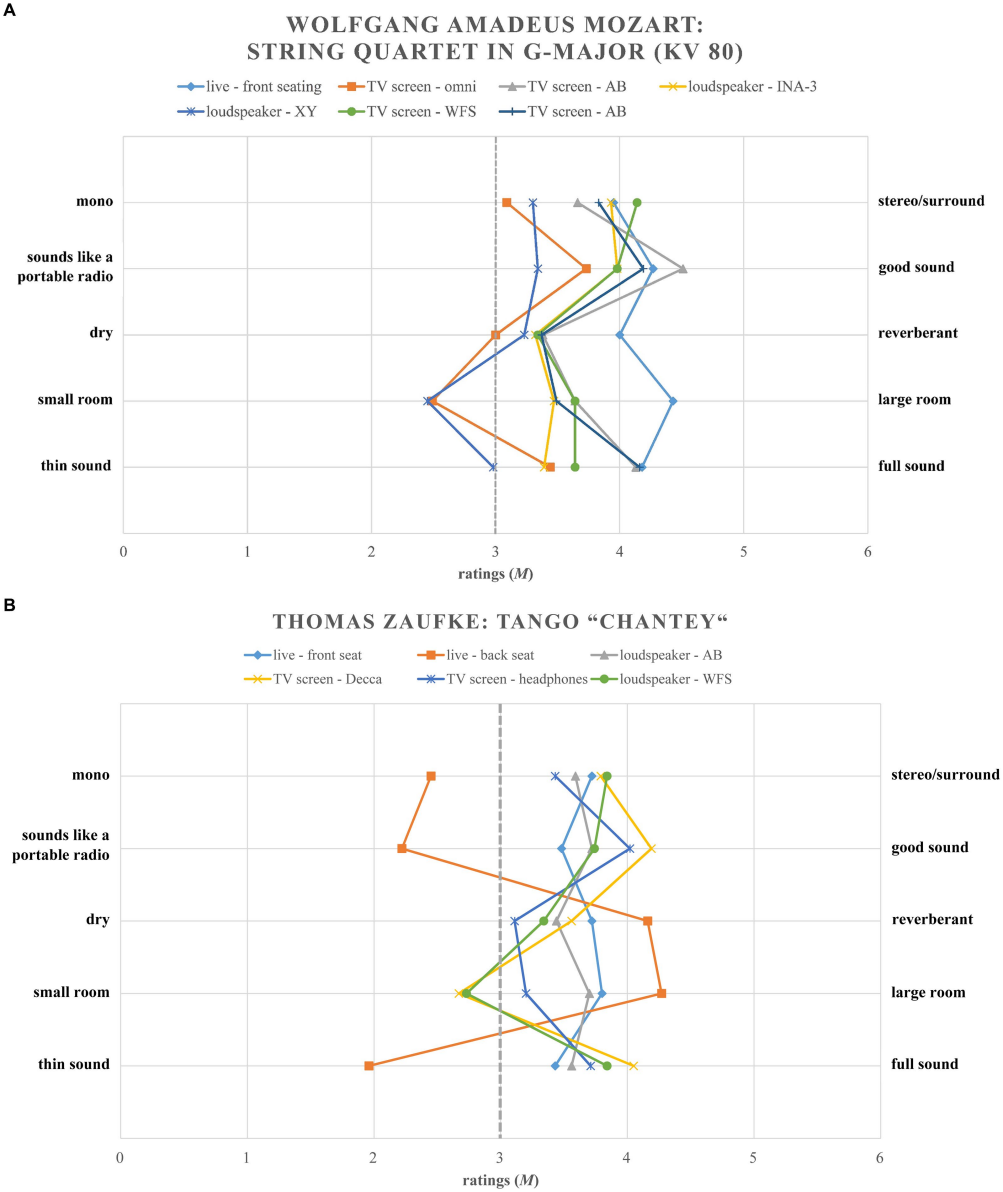
**(A)** Mean ratings (*M*) of the Mozart stimuli for items of the created subscales *sound quality* and *room size* over all stimuli. **(B)** Mean ratings (*M*) of the Zaufke stimuli for items of the created subscales *sound quality* and *room size* over all stimuli.

For the development of the subscales, the findings of the PCAs of the first study were used and supplemented with theoretical foundations, discriminatory power and Cronbach’s Alpha. Figurative representations of the ratings of the stimuli served as additional interpretation aid. For example, it can be seen, that the item *thin sound – full sound* is a much better fit for the subscale *sound quality* (*mono – stereo/surround* and *sounds like a portable radio – good sound*) than for the subscale *room size* (*dry – reverberant* and small *room – large room*). This is particularly clear for stimulus “Zaufke. Live – rear seating” (*cf.* Figures 3A,B).

**Figure 4:**
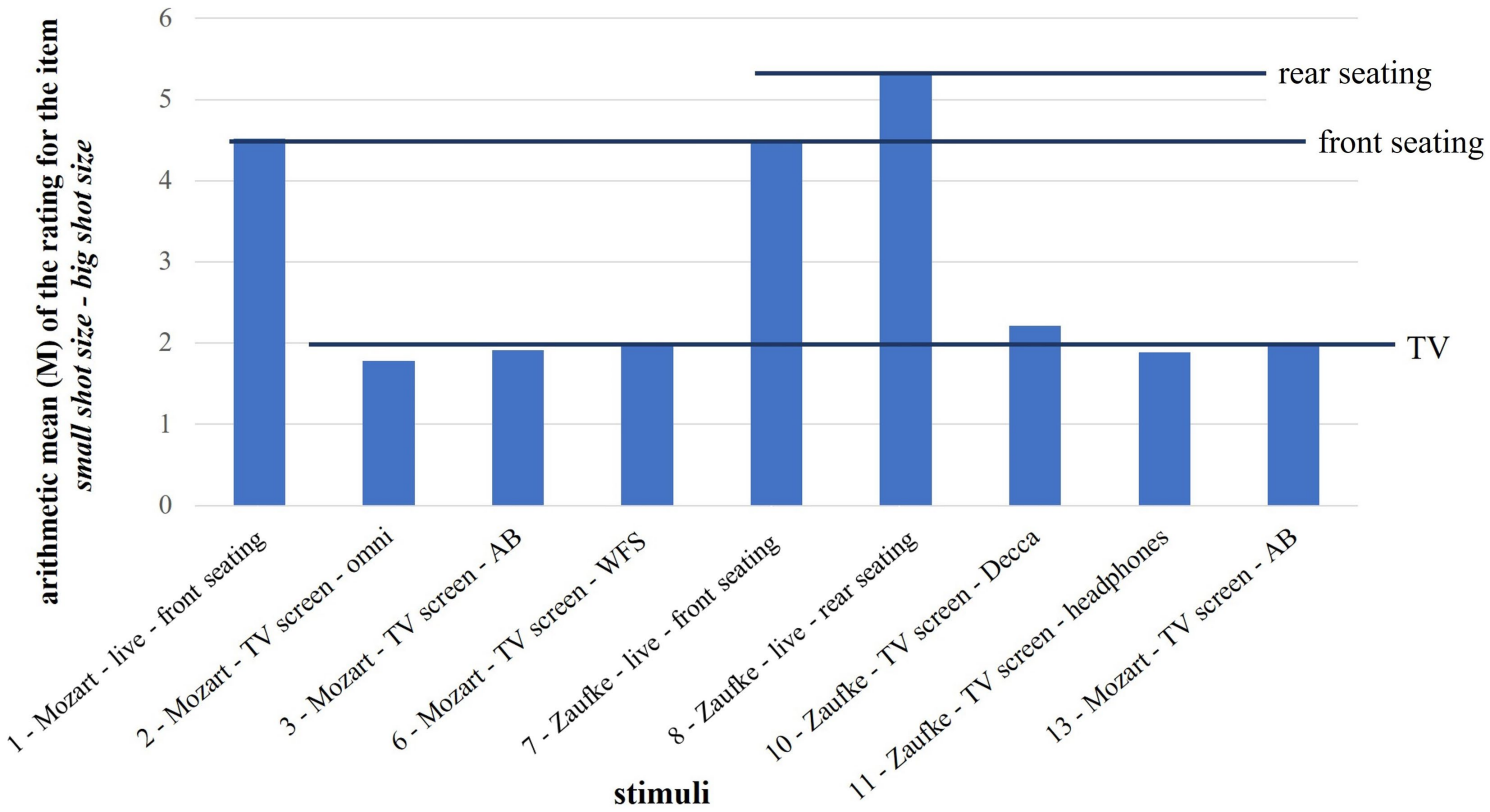
Arithmetic mean (*M*) of the rating for the item s*mall shot size – big shot size.*

The formulations of some items were adjusted. For example, the formulation *small shot size*^2^ *– big shot size* was changed into *improved sight due to shot size – hindered sight due to shot size* in order to achieve less trivial results. As can be seen in Figure 4, the item only divided the stimuli into “front seating,” “rear seating” and “TV.”

Some items were not assigned to a subscale but kept as single items.

#### 3.2.5 The semantic differential – Giessen Music Mediation Inventory (GMMI)

The analysis terminated in the semantic differential with 60 items consisting of 18 subscales and nine single items shown in Table 6.

**Table 6:** The Giessen Music Mediation Inventory (GMMI) with Cronbach’s Alpha and discriminatory power for the categories *acoustic*, *visual*, *interaction of acoustic and visual* and *general assessment*.

Scale name (German original), Cronbach’s Alpha	Item (German original)	Discriminatory power
Acoustic		
Sound quality (Klangqualität), *α* = 0.847	Mono – stereo/surround (Mono – Stereo/Surround)	0.879
	Sounds like a portable radio – good sound (high-end) (Kofferradioklang – guter Klang [High-End])	0.753
	Thin sound – full sound (Dünner Klang – voller Klang)	0.733
Room size (Raumgröße), *α* = 0.886	Dry – reverberant (Trocken – hallig)	0.802
	Small room – large room (Kleiner Raum – großer Raum)	0.802
Vitality (Vitalität), *α* = 0.896	Cold – warm (Kalt – warm)	0.795
	Lifeless – lively (Leblos – lebhaft)	0.720
	Uninspired – spirited (Uninspiriert – geistvoll)	0.778
	Sterile – full of life (Steril – voll Leben)	0.798
Hearing transparency (Durchhörbarkeit), *α* = 0.636	Instruments can be poorly localized – instruments can be well localized (Instrumente schlecht zu orten – Instrumente gut zu orten)	0.488
	Obscure – transparent (Undurchsichtig – transparent)	0.488
Pitch (Tonhöhe), *α* = 0.771	Bright – dark (Hell – dunkel)	0.628
	Highs dominate – lows dominate (Höhen dominieren – Tiefen dominieren)	0.628
Balance of the ensemble (Ensemblebalance), *α* = 0.770	Musicians at different distances – musicians at same distance (Musizierende unterschiedlich weit entfernt – Musizierende in gleicher Entfernung)	0.515
	Frequencies not balanced – frequencies balanced (Frequenzen nicht ausgeglichen – Frequenzen ausgeglichen)	0.792
	Voices not balanced – good balance of the voices (Stimmen nicht ausgeglichen – gute Balance der Stimmen)	0.531
Balance of the ensemble – direction (Ensemblebalance – Richtung), *α* = 0.650	Single (instrumental) voice(s) in the foreground – single (instrumental) voice(s) in the background (Einzelne (Instrumental-) Stimme(n) im Vordergrund – einzelne (Instrumental-)Stimme(n) im Hintergrund)	0.501
	main voice overrepresented – main voice underrepresented (Hauptstimme/ Melodiestimme überrepräsentiert – Hauptstimme/ Melodiestimme unterrepräsentiert)	0.501
Naturalness (Natürlichkeit), *α* = 0.862	Edited – natural (Bearbeitet – natürlich)	0.759
	Artifical – authentic (Künstlich – authentisch)	0.759
Localization of the sound (Klanglokalisation) – single items	Sound from the left – sound from the right (Klang von links – Klang von rechts)	–
	Sound from the front – sound from the back (Klang von vorne – Klang von hinten)	–
	Music plays in the head – music plays in the room (Musik spielt im Kopf – Musik spielt im Raum)	–
	Sound from the top – sound from the bottom (Klang von oben – Klang von unten)	–
Loudness (Lautheit)	Loud – quiet (Laut – leise)	–
Distance (Entfernung)	(Acoustically) near – (acoustically) distant ([Akustisch] nah – [akustisch] entfernt)	–
Skills of the musicians (Fähigkeiten der MusikerInnen), scale heavily revised	With ease – strained (Mit Leichtigkeit – angestrengt)	–
	Professional musicians – amateur musicians (ProfimusikerInnen – AmateurmusikerInnen)	–
Visual		
Coloring (Farbgebung), *α* = 0.837	Cold light – warm light (Kaltes Licht – warmes Licht)	0.719
	Blueish colors – yellowish colors (Bläuliche Farben – gelbliche Farben)	0.719
Entertainment (Unterhaltung), *α* = 0.720	Reserved gestures – exaggerated gestures (Zurückhaltende Gestik – übertriebene Gestik)	0.466
	Steady colors – varying colors (Farblich gleichförmig – farblich abwechslungsreich)	0.500
	Static picture – moving picture (Statisches Bild – bewegtes Bild)	0.578
	Reticent picture – entertaining picture (Bild zurückgenommen – Bild unterhaltsam)	0.711
Aura of the musicians (Ausstrahlung der MusikerInnen), *α* = 0.769	Involved aura of the musicians – distanced aura of the musicians (Involvierte Ausstrahlung der MusikerInnen – MusikerInnen wirken distanziert)	0.626
	Musicians appear present – musicans appear lost (MusikerInnen wirken präsent – MusikerInnen wirken verloren)	0.626
Picture dramaturgy (Bilddramaturgie), *α* = 0.607	Interactions clearly visible – interaction poorly visible (Interaktionen gut sichtbar – Interaktionen schlecht sichtbar)	0.437
	Improved sight due to shot size – hindered sight due to shot size (Bessere Sicht durch Bildausschnitt – behinderte Sicht durch Bildausschnitt)	0.437
Naturalness (Natürlichkeit)	Artifical environment – natural environment (Künstliche Umgebung – natürliche Umgebung)	–
Distance (Entfernung)	(Visually) near – (visually) distant ([Optisch] entfernt – [optisch] nah)	–
Interaction of acoustic and visual		
Fit of sound and picture (Ton-Bild-Passung), *α* = 0.864	Mix (sound) is adjusted to the image – mix (sound) ignores the image (Klangliche Mischung ist bildbezogen – klangliche Mischung lässt Bild außen vor)	0.675
	Sound fits well with the image – sound fits poorly with the image (Ton passt gut zum Bild – Ton passt schlecht zum Bild)	0.821
	Expectations which are created by the images are fulfilled – expectations which are created by the images are not fulfilled (Durch Bild erzeugte Erwartungen werden erfüllt – durch Bild erzeugte Erwartungen werden nicht erfüllt)	0.747
	The interaction between image and sound clarifies – the interaction between image and sound confuses (Zusammenspiel von Bild und Ton ist hilfreich – Zusammenspiel von Bild und Ton verwirrt)	0.628
Number of musicians (Musikeranzahl)	One seems to hear more musicians than there actually are – one can hear the exact number of musicians (Man glaubt mehr MusikerInnen zu hören als da sind – man glaubt weniger MusikerInnen zu hören als da sind)	–
Visual and acoustic distance (Optische und akustische Entfernung)	Visually and acoustically at different distances – visually and acoustically at the same distance (Optisch und akustisch unterschiedlich weit entfernt – optisch und akustisch in gleicher Entfernung)	–
General assessment		
Emotional stimulation (Emotionale Anregung), *α* = 0.923	Unsatisfactory – enthralls me (Überzeugt mich nicht – reißt mich mit)	0.784
	Neutral feelings – touches the soul (Gefühlsneutral – geht direkt in die Seele)	0.860
	Does not reach me emotionally – evokes intense emotions (Erreicht mich emotional kaum – wirkt auf mich emotional intensiv)	0.910
Immersion (Immersion), *α* = 0.898	Pulls me in – does not pull me in (Zieht mich rein – wenig einbezogen)	0.769
	involved – reserved (Involviert – Distanziert)	0.846
	I feel like I am there – I feel far away (Fühle mich vor Ort – fühle mich außen vor)	0.792
Inner peace (Innere Ruhe), *α* = 0.853	Cozy – stressful (Gemütlich – stressig)	0.745
	Calming – stirring (Beruhigend – aufwühlend)	0.745
Value judgment (Werturteil), *α* = 0.911	Appealing – boring (Reizvoll – Langweilig)	0.762
	Pleasant – unpleasant (Angenehm – unangenehm)	0.833
	I like – I dislike (Gefällt mir – gefällt mir nicht)	0.850
	Beautiful – ugly (Schön – hässlich)	0.879
Publicity (Öffentlichkeit)	Private – public (Privat – öffentlich)	–
Attention (Aufmerksamkeit)	Causes intense listening – seems like background music (Bewirkt intensives Zuhören – wirkt wie Hintergrundmusik)	–

#### 3.2.6 Comparison of different presentation modes

**Figure 5:**
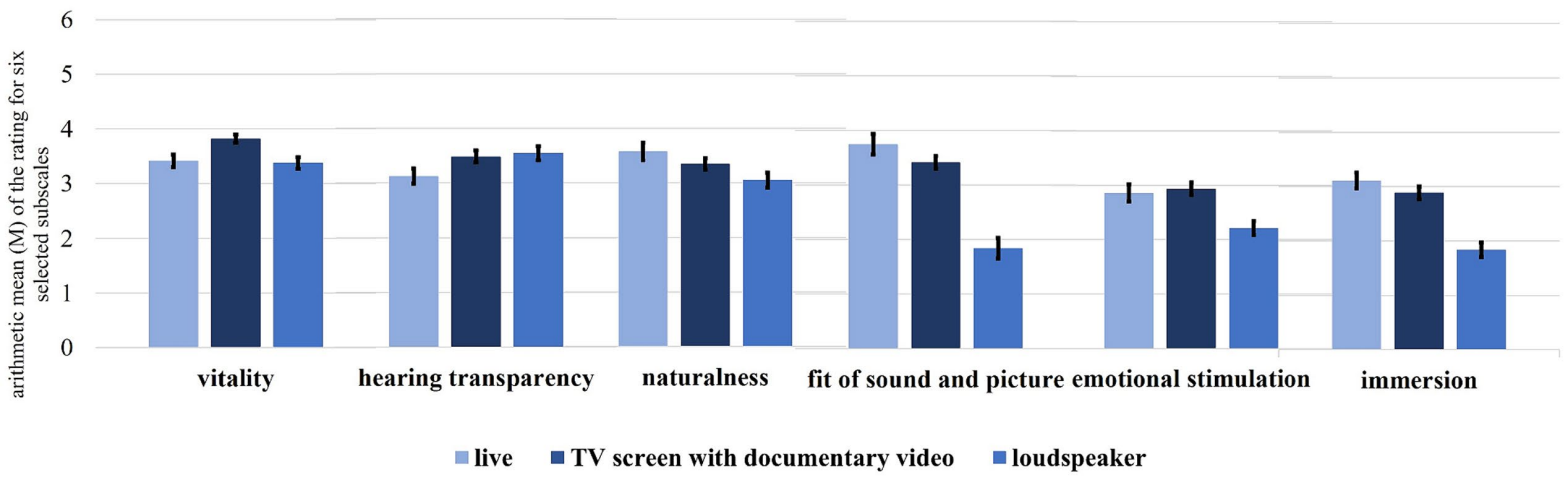
Arithmetic mean (*M*) with standard error of the rating for six selected subscales, comparing three 3D simulations types (live, TV screen with documentary video and loudspeaker).

In the follow-up to the creation of the GMMI, some of subscales were examined with regard to the differences between the presentation modes “live,” “TV screen with documentary video” and “loudspeaker.” Here, the results of the ANOVAs over the subscales *vitality*, *hearing transparency*, *naturalness*, *fit of sound and picture*, *emotional stimulation* and *immersion* will be presented. Figure 5 shows these ratings, while Table 7 shows the results of the performed ANOVAs.

**Table 7:** ANOVA results for the audio-visual presentation modes “live,” “TV screen with documentary video” and “loudspeaker”: number (*n*), ANOVA results, *p*-value and effect size Cohen’s ƒ.

	*n*	ANOVA	*p*	ƒ
Hearing transparency	45	*F* (2, 88) = 4.77	0.011	0.3296
Naturalness	45	*F* (2, 88) = 5.93	0.004	0.3675
Vitality	45	*F* (2, 88) = 13.63	<0.001	0.5573
Fit of sound and picture	41	*F* (2, 80) = 47.37	<0.001	1.0878
Emotional stimulation	45	*F* (2, 88) = 12.95	<0.001	0.5419
Immersion	45	*F* (1.7, 72.8) = 28.5	<0.001	0.8046

All of the subscales reached the significance level, so post-hoc tests with Bonferroni-Holm correction were used to find the relevant differences. For *vitality*, the post-hoc tests show a difference between “loudspeaker” and “TV screen with documentary video” (*p* < 0.001) as well as between “TV screen with documentary video” and “live” (*p* < 0.001), but no difference between “loudspeaker” and “live” (*p* = 0.714). This indicates an influence of picture dramaturgy on the *vitality* rating.

For *hearing transparency*, the presentation mode “live” reached the lowest value; *hearing transparency* was also significant between “live” and “loudspeaker” (*p* = 0.035) and “live” and “TV screen with documentary video” (*p* = 0.035), but not between the two medial presentations (*p* = 0.647). It is quite astonishing that a live presentation impairs instrumental localization and sound transparency and that sight does not make a difference.

The subscales *naturalness*, *fit of sound and picture*, *emotional stimulation* and *immersion* all reached the lowest values for the stimuli in the “loudspeaker”-presentation mode. They reached significant (or at least almost significant) values between “loudspeaker” and “TV screen with documentary video” (*p*_naturalness_ = 0.052, *p*_fit of sound and picture_ < 0.001, *p*_emotional stimulation_ < 0.001 and *p*_immersion_ < 0.001) and between “loudspeaker” and “live” (*p*_naturalness_ = 0.013, p_fit of sound and picture_ < 0.001, *p*_emotional stimulation_ < 0.001 and *p*_immersion_ < 0.001), but not between “TV screen with documentary video” and “live”—which is interesting as it indicates that a presentation’s being live or medial is less relevant than if it involves an visual performance of the musicians or not.

## 4 Discussion

Our two studies applied stimuli that comprised not real, but rather simulated performances in various live and medially presented listening situations. This was necessary to ensure that all stimuli were based on the same sound recordings of the two pieces of music and the same video footage. Only in this way could differences in perception, experience and aesthetic judgment be attributed solely to various presentation modes. In Study 1, we captured all dimensions relevant for assessing differences of these different presentation modes. A qualitative survey was therefore conducted and evaluated using a mixed-methods approach. In Study 2, the dimensions of perception, experience and aesthetic judgment were evaluated using a different sample, but including a selection of previously used stimuli. All items were examined by means of an item analysis, descriptive statistics and analyses of variance with regard to their understandability and their power to distinguish between different stimuli. If discrepancies occurred, these too were analyzed, and the items in question were either revised or eliminated. For the sample, prior musical experience had very little influence on the ratings. This is a desirable result as the aim of this study was to create a questionnaire that could be used independently of deeper understanding of the subject matter. If the fit was sufficient, the items were summarized in thematic scales. Items that could not be assigned to a scale were checked for their relevance and either eliminated or retained as single items. In only a few cases did the formulations have to be adjusted afterwards when it seemed meaningful to create a more specific item. In total, only a small number of items had to be eliminated or changed. This is a satisfactory result as it confirms the results of the first study.

Nevertheless, another evaluation of the GMMI would be desirable. As both studies used the same stimuli, a study with new stimuli and perhaps actual live performances as compared with medial presentations, would be ideal. By now, the GMMI is limited to the German language as the vocabulary was explicitly designed for German linguistic customs.

With 60 items, the GMMI is a rather long semantic differential. It should therefore be kept in mind that the questionnaire consists of four categories, so a modular use is conceivable, too. Depending on the research aim or the presented stimuli, not all items will always fit. If for example only aural presentation modes are to be examined, items for the categories “visual” and “interaction between acoustic and visual” cannot be answered reasonably.

There are also some limitations resulting from the original choice of stimuli. At the beginning, the decision was made to exclude moving images in the form of music videos with a narrative or abstract concept that might have added another level of meaning beyond the mere presentation of musicians. For this kind of music video, which is more common in the pop music market, an evaluation category is missing.

Taking into account the natural frequency range of the selected pieces of music, it made sense not to use an additional subwoofer in this study. However, this could become relevant for stimuli comprising other musical genres that are usually accompanied by more physical experiences due to deep bass vibrations (e.g., in rock music).

Furthermore, the social component is nearly missing, as this would have led to a large number of hardly controllable confounding variables. As in particular, the social component of music listening has proved to be a large area of research (e.g., Roose and Vander Stichele, 2010; Brown and Knox, 2017; Swarbrick et al., 2019 or Brown and Krause, 2020), this limitation seems bearable. At the same time, the absence of social variables such as a visible audience is a strength of this study, too, as only in this way was it possible to really capture perceptual differences unbiased by these visual components. Under these circumstances it is all the more remarkable that our second study provides evidence that listeners feel significantly higher emotional stimulation and immersion when performing musicians are presented visually – whether live or on TV – as compared with by loudspeaker alone. This corresponds with the idea of an emergent property created by the interaction between the two sensory modalities conveyed in musical performances and perceived by audience members (Chapados and Levitin, 2008).

Hearing ability, which features in the second study, was only checked by self-report. As some participants were of older age it is rather unlikely that there were no hearing constraints at all. Nonetheless, even the elderly people passed the reliability check, and by including a broader age range a more realistic image of music listeners could be created – especially for classical music, where the audience consists of many different groups of people (Gembris and Menze, 2021). However, subsequent studies might include a brief hearing test.

With an average duration of 2 h, the second study was quite long, so that it cannot be excluded that some participants had signs of fatigue and declining concentration. The long duration was necessary to cover a broad range of different live and media perception situations so that no possible differences were lost in the semantic differential. An attempt was made to compensate this by randomizing the stimuli. A shorter duration would be desirable for future studies.

For future research, the GMMI can be used, for example, to examine the influence of the visual on the acoustic judgment and vice versa, simply by varying a single parameter. Progress in the field of virtual reality will probably lead to live simulations that become increasingly better. Here, the GMMI could be used to compare a simulation with a live performance. This could also be an interesting topic for the field of consumer research. Rondán-Cataluña and Martín-Ruiz (2010) as well as Mortimer et al. (2012) did not find any negative effects of the prevalence of aural media products on the consumption of live concerts. Taking into account the virtual reality concerts with ABBA-Avatars performing their new album and old hits, which began in 2022 (Woolley and Collins, 2019; Hughes, 2020), it would be possible to study whether live simulations or audio-visual media products have the potential to compete with live concerts.

As we saw in our comparison of different presentation modes, there was no clear ranking between “loudspeaker,” “TV screen with documentary video” and “live.” Especially between the simulations of medial presentations with moving pictures and the live concert simulations, there were often only small differences that did not reach the significance level. It seems likely that other studies, such as that of Shoda et al. (2016), who examined live music in contrast to aurally presented music, would have led to different results if they had included audio-visually presented music.

## 5 Conclusion

What our two studies have achieved is the wide-ranging acquisition of assessment dimensions and the 27 subscales, which document a so far unprecedented number of differences in the perception, experience and aesthetic judgment of live or medially presented music. More research is nonetheless needed to build up a more nuanced picture of the ways in which listeners evaluate pieces of music depending on presentation mode.

The research area of medially presented music with and without a visual component could be extended with the use of different recording technology and quality. For example, a study that led to the result that the visual component had a positive influence could be replied with different acoustic qualities. An audio-visual stimulus combined with sound of low quality can be contrasted to an only acoustic stimulus with high quality sound. The GMMI can be used to identify all expected as well as unexpected parameters that are influenced by these variations.

## Data availability statement

The original contributions presented in the study are included in the article/Supplementary material, further inquiries can be directed to the corresponding author.

## Ethics statement

Ethical approval was not required for the studies involving humans because the study was conducted in full accordance with the Ethical Guidelines of the German Association of Psychologists (DGPs) and the German Association of Psychologists (BDP) as well as the Ethical Principles of Psychologists and Code of Conduct of the American Psychological Association (APA). These guidelines suggest that for the type of research reported here, a formal ethics approval is not necessary. The present study only used completely anonymous questionnaires. All data of the MPI participant database was stored separately from the survey data. The studies were conducted in accordance with the local legislation and institutional requirements. The participants provided their written informed consent to participate in this study.

## Author contributions

LM: Conceptualization, Data curation, Formal analysis, Investigation, Methodology, Software, Supervision, Validation, Visualization, Writing – original draft, Writing – review & editing. CB: Conceptualization, Formal analysis, Investigation, Methodology, Project administration, Supervision, Writing – original draft, Writing – review & editing. AL: Writing – review & editing, Supervision, Resources, Methodology, Formal analysis, Data curation, Conceptualization. MW-F: Project administration, Resources, Writing – review & editing.

## Footnotes

^1^The frequency response of the headphones was electronically compensated within a frequency range of 50–20,000 Hz. The use of an additional subwoofer was omitted with regard to the lowest frequencies of the selected musical pieces.

^2^Other possible translations for the German word “Bildausschnitt” are “picture section,” “image detail” or “section of the image” meaning what part of a scenery can be seen. The German term is not limited to a cinematic context.

## References

[ref1] Brown S. C. Knox D. (2017). Why go to pop concerts? The motivations behind live music attendance. Music. Sci. 21, 233–249. doi: 10.1177/1029864916650719

[ref2] Brown S. C. Krause A. E. (2020). Freedom of choice: examining music listening as a function of favorite music format. Psychomusicology 30, 88–102. doi: 10.1037/pmu0000254

[ref3] Bullerjahn C. Hantschel F. (2018). “Musik im audiovisuellen Kontext: Film, Fernsehen, Video(spiel) [Music in the audio-visual context: film, television and video (game)]” in Handbuch Musikpsychologie [Handbook music psychology]. eds. Lehmann A. C. Kopiez R. (Bern: Hogrefe), 273–290.

[ref4] Chapados C. Levitin D. J. (2008). Cross-modal interactions in the experience of musical performances: physiological correlates. Cognition 108, 639–651. doi: 10.1016/j.cognition.2008.05.008, PMID: 18603233

[ref5] Coutinho E. Scherer K. R. (2017). The effect of context and audio-visual modality on emotions elicited by a musical performance. Psychol. Music 45, 550–569. doi: 10.1177/0305735616670496, PMID: 28781419 PMC5519088

[ref6] Elste M. (1992). “Technische Reproduktion [Technical reproduction]” in Neues Handbuch der Musikwissenschaft [New handbook on musicology]. eds. Binkley T. Danuser H. (Laaber: Laaber-Verl), 401–444.

[ref7] Finnäs L. (2001). Presenting music live, audio-visually or aurally – does it affect listenersʼ experiences differently? Br. J. Music Educ. 18, 55–78. doi: 10.1017/S0265051701000146

[ref8] Flink S. F. (1990). The effect of live music versus tape-recorded music on participation rates of two populations. Honors thesis 1610. Kalamazoo: Western Michigan University. Available at: https://scholarworks.wmich.edu/honors_theses/1610 (Accessed April 26, 2022).

[ref9] Gembris H. Menze J. (2021). “Between audience decline and audience development: perspectives on professional musician, music education, and cultural policy” in Classical concert studies. A companion to contemporary research and performance. ed. Tröndle M. (New York, Abingdon: Routledge), 211–226.

[ref10] Greb F. Schlotz W. Steffens J. (2018). Personal and situational influences on the functions of music listening. Psychol. Music 46, 763–794. doi: 10.1177/0305735617724883

[ref11] Griffiths N. K. Reay J. L. (2018). The relative importance of aural and visual information in the evaluation of Western canon music performance by musicians and nonmusicians. Music. Percept. 35, 364–375. doi: 10.1525/mp.2018.35.3.364

[ref12] Horn M. (2013). *Vergleichende Untersuchung live aufgeführter und medial reproduzierter Musik mittels einer immersiven Hörversuchsumgebung [Comparative study of live performed and medial reproduced music via an immersive listening context]*. Master’s Thesis. Technische Universität Berlin. Available at: http://www2.users.ak.tu-berlin.de/akgroup/ak_pub/abschlussarbeiten/2013/HornMichael_MagA.pdf (Accessed April 26, 2022).

[ref13] Horn M. Lindau A. Maempel H. J. Weinzierl S. (2015). “Livekonzert und Medienmusik: immersive opto-akustische simulation als Werkzeug der Musik- und Medienrezeptionsforschung [Live concert and media music: immersive opto-acoustic simulations as a tool for music and media perception research]” in Musikpsychologie – Jahrbuch der Deutschen Gesellschaft für Musikpsychologie [Music psychology – Yearbook of the German Society for Music Psychology]. eds. Auhagen W. Bullerjahn C. Von Georgi R., *Vol.* 25 (Göttingen: Hogrefe), 246–249. doi: 10.23668/psycharchives.2839

[ref14] Hughes A. (2020). “Death is no longer a Deal breaker. The hologram performer in live music” in The future of live music. eds. Marierska E. Gillon L. Rigg T. (New York, London: Bloomsbury Academic), 114–128.

[ref15] Kelly G. A. (1991). *The psychology of personal constructs*. Vol 1: A theory of personality. London: Routledge.

[ref16] Li S. Timmers R. Wang W. (2021). The communication of timbral intentions between pianists and listeners and its dependence on auditory-visual conditions. Front. Psychol. 12:717842. doi: 10.3389/fpsyg.2021.717842, PMID: 34621217 PMC8491637

[ref17] Lindau A. (2010). “Zu den Dimensionen des Unterschieds live aufgeführter und reproduzierter Musik. Ergebnisse einer qualitativ-quantitativen Umfragestudie [On the dimensions of live performed and reproduced music. Results of a qualitative-quantitative survey study]” in Fortschritte der Akustik – DAGA 2010 [Progress of acoustics – DAGA 2010]. eds. Möser M. Schulte-Fortkamp B. Ochmann M. (Berlin: Deutsche Gesellschaft für Akustik E.V.), 609–610.

[ref18] Lindau A. (2014). *Binaural resynthesis of acoustical environments. Technology and Perceptual Evaluation*. Dissertational Thesis. Technische Universität Berlin. Available at: https://api-depositonce.tu-berlin.de/server/api/core/bitstreams/76925df3-459b-4275-aa93-91ddec78564f/content (Assessed March 12, 2024).

[ref19] Lipscomb S. D. Kerins M. (2004). “An empirical investigation into the effect of presentation mode in the cinematic and music listening experience” in Proceedings of the 8^th^ international conference on music perception & cognition. eds. Lipscomb S. Ashley R. Gjerdingen R. Webster P. (Sydney: Causal Productions), 528–533.

[ref20] Maempel H.-J. (2008). “Medien und Klangästhetik [Media and sound aesthetics]” in Musikpsychologie. Das neue Handbuch [Music psychology. The new handbook]. eds. Bruhn H. Kopiez R. Lehmann A. C. (Reinbek: Rowohlt), 231–252.

[ref21] Maempel H.-J. Weinzierl S. Kaminski P. (2008). “Audiobearbeitung [Audio editing]” in Handbuch der Audiotechnik [Handbook on audio engineering]. ed. Weinzierl S.. 2nd ed (Berlin and Heidelberg: Springer), 719–785.

[ref22] Mayring P. (2015). Qualitative Inhaltsanalyse. Grundlagen und Techniken [Qualitative content analysis. Basics and techniques]. 12th Edn. Weinheim and Basel: Beltz.

[ref23] Moldrzyk C. Lenz T. Weinzierl S. (2005). “Perzeptive evaluation binauraler Auralisationen [Perceptual evaluation of binaural auralizations]” in Fortschritte der Akustik – DAGA 2005 [Progress of acoustics – DAGA 2005]. eds. Fastl H. Fruhmann M. (Berlin: Deutsche Gesellschaft für Akustik e. V.), 545–546.

[ref24] Mortimer J. H. Nosko C. Sorensen A. (2012). Supply responses to digital distribution: recorded music and live performances. Inf. Econ. Policy 24, 3–14. doi: 10.1016/j.infoecopol.2012.01.007

[ref25] Pitts S. (2014). “Musical, social and moral dilemmas: investigating audience motivations to attend concerts” in Coughing and clapping: Investigating audience experience. eds. Burland K. Pitts S. (Farnham: Ashgate), 21–34.

[ref26] Platz F. Kopiez R. (2012). When the eye listens. A Meta-analysis of how audio-visual presentation enhances the appreciation of music performance. Music Percept. 30, 71–83. doi: 10.1525/mp.2012.30.1.71

[ref27] Rocholl P. (1976). “Fragen der unterschiedlichen Vermittlung von Musikwerken in den Medien – Gründe, Tendenzen, Auswirkungen [Questions on different mediations of musical compositions in the media – reasons, tendencies, effects]” in Musik in den Massenmedien Rundfunk und Fernsehen. Perspektiven und Materialien [Music in the mass media broadcast and television. Perspectives and materials]. ed. Schmidt H.-C. (Mainz: Schott), 74–90.

[ref28] Rondán-Cataluña F. J. Martín-Ruiz D. (2010). Customersʼ perceptions about concerts and CDs. Manag. Decis. 48, 1410–1421. doi: 10.1108/00251741011082152

[ref29] Roose H. Vander Stichele A. (2010). Living room vs. concert hall: patterns of music consumption in Flanders. Soc. Forces 89, 185–207. doi: 10.1353/sof.2010.0077

[ref30] Rosenberger M. Freitag M. (2009). “Repertory grid” in Handbuch Methoden der Organisationsforschung. Quantitative und Qualitative Methoden [Handbook methods of organizational research. Quantitative and qualitative methods]. eds. Kühl S. Strodtholz P. Taffertshofer A. (Wiesbaden: VS Verlag für Sozialwissenschaften), 477–496.

[ref31] Schaal N. K. Bauer A. K. R. Müllensiefen D. (2014). Der Gold-MSI. Replikation und Validierung eines Fragebogeninstrumentes zur Messung Musikalischer Erfahrenheit anhand einer deutschen Stichprobe [The Gold-MSI inventory. Replication and validation of a tool for evaluating musical sophistication with a German sample]. Music. Sci. 18, 423–447. doi: 10.1177/1029864914541851

[ref32] Schäfer T. Sedlmeier P. (2018). “Musik im Alltag: Wirkungen, Funktionen und Präferenzen [Music in everyday life: effects, functions and preferences]” in Handbuch Musikpsychologie [Handbook music psychology]. eds. Lehmann A. C. Kopiez R. (Bern: Hogrefe), 247–271.

[ref33] Shoda H. Adachi M. Umeda T. (2016). How live performance moves the human heart. PLoS One 11:e0154322. doi: 10.1371/journal.pone.0154322, PMID: 27104377 PMC4841601

[ref34] Strauss A. L. (1994) Grundlagen qualitativer Sozialforschung. Datenanalyse und Theoriebildung in der empirischen soziologischen Forschung [Fundamentals of qualitative social research. Data analysis and theory building in empirical sociological research]. 2nd Edn. München: Fink.

[ref35] Swarbrick D. Bosnyak D. Livingstone S. R. Bansal J. Marsh-Rollo S. Woolhouse M. H.. (2019). How live music moves us: head movement differences in audiences to live versus recorded music. Front. Psychol. 9:2682. doi: 10.3389/fpsyg.2018.02682, PMID: 30687158 PMC6336707

[ref36] Vuoskoski J. K. Thompson M. R. Clarke E. F. Spence C. (2014). Crossmodal interactions in the perception of expressivity in musical performance. Atten. Percept. Psychophys. 76, 591–604. doi: 10.3758/s13414-013-0582-2, PMID: 24233641

[ref37] Vuoskoski J. K. Thompson M. R. Spence C. Clarke E. F. (2016). Interaction of sight and sound in the perception and experience of musical performance. Music. Percept. 33, 457–471. doi: 10.1525/MP.2016.33.4.457

[ref38] Wald-Fuhrmann M. Egermann H. Czepiel A. O’Neill K. Weining C. Meier D.. (2021). Music listening in classical concerts: theory, literature review, and research program. Front. Psychol. 12:1324. doi: 10.3389/fpsyg.2021.638783, PMID: 33986708 PMC8110713

[ref39] Woolley S. I. Collins T. (2019). User experience and engagement in the reality–Virtuality continuum: a special issue guest editorial. Presence Teleop. Virt. 28, 203–206. doi: 10.1162/pres_e_00348

